# Noncontrast Computed Tomography Parameters for Predicting Shock Wave Lithotripsy Outcome in Upper Urinary Tract Stone Cases

**DOI:** 10.1155/2018/9253952

**Published:** 2018-12-02

**Authors:** Shimpei Yamashita, Yasuo Kohjimoto, Yuya Iwahashi, Takashi Iguchi, Satoshi Nishizawa, Kazuro Kikkawa, Isao Hara

**Affiliations:** Department of Urology, Wakayama Medical University, 811-1 Kimiidera, Wakayama 641-0012, Japan

## Abstract

Kidney stones are a major public health concern with continuously increasing worldwide prevalence. Shock wave lithotripsy (SWL) is the first line treatment choice for upper urinary tract calculi with ureteroscopy and has advantages of safety and noninvasiveness, but the treatment success rate of SWL is lower than that of other therapies. It is therefore important to identify predictive factors for SWL outcome and select a suitable treatment choice for patients with upper urinary tract calculi. In recent years, computed tomography (CT) has become the gold standard for diagnosis of upper urinary tract calculi. Several factors based on CT images, including skin-to-stone distance, mean stone density, stone heterogeneity index, and variation coefficient of stone density, have been reported to be useful for predicting SWL outcome. In addition, a new method of analysis, CT texture analysis, is reportedly useful for predicting SWL outcomes. This review aims to summarize CT parameters for predicting the outcome of shock wave lithotripsy in stone cases in the upper urinary tract.

## 1. Introduction

Kidney stones are a major public health concern with continuously increasing prevalence [1]. In developed countries, the prevalence has increased from 5% in 1994 to approximately 10% in the 2000s [2].

The first line treatment choice for upper urinary tract calculi is currently shock wave lithotripsy (SWL). While it has advantages of safety and low-invasiveness, its treatment success rate is lower than that of other therapies [3]. Predictive factors for SWL outcome must be identified and suitable treatment choice for patients with upper urinary tract calculi must be selected.

In recent years, noncontrast computed tomography (CT) has become the gold standard for diagnosis of upper urinary tract calculi, and several factors based on CT images have been reported to be useful for prediction of SWL outcome in addition to stone size and location. Here, we review the usefulness of these predictive factors.

## 2. Stone Size/Volume

Although previous studies have shown that stone size is important factor for predicting SWL outcome and stones over 2 cm are associated with an inferior outcome [4–7], the imaging modality used for evaluating stone size varies among studies [8]. The difference of imaging modalities can lead to the discrepancies in the measurement of the stone dimensions [8]. A plain abdominal film (KUB) is generally viewed only in the coronal plane. In addition, a magnification error with KUB can lead to an increase in stone size by 20% [9]. Ultrasonography (US) makes it possible to measure the stone dimensions in any plane; however, the reproducibility of stone size measurements can be low because US does not offer the fixed planes like KUB or CT. US has also been shown to overestimate the stone size compared with CT, especially for small stones ≤ 5 mm [10].

Compared with KUB or US, the stone size measurements for CT images have been reported to be more accurate and reproducible with no magnification error and less observer bias [8]. Using coronal reconstruction, CT images can provide the measurement of cephalocaudal dimensions in addition to axial stone images. It has been reported that coronal CT images provide a different impression of stone size and should also be used to measure stone size more accurately [11]. Moreover, the previous study has shown that magnified bone windows constitute more accurate method of stone measurements in vitro and in vivo than standard soft tissue windows [12]. Therefore, the routine use of bone windows and the measurement of stone dimensions in the axial and coronal dimensions are recommended to accurately access the stone size [8].

Using three-dimensional analyzing software, CT images can provide information about stone volume. It has also been reported that stone volume is a better predictor of SWL outcome than stone length or width [13]. Future large-scale studies are required to decide the optimal cutoff value for stone volume.

## 3. Stone Location

Stone location is also an important factor for predicting SWL outcome. The previous large-scale study has reported that the treatment success rate in ureteral stone cases is higher than that in renal stone cases [6]. In addition, it has also shown that the stone-free rate in lower pole stone cases is lower than that in renal pelvic, upper pole and ureteropelvic junction cases [14–16]. We can obtain the information about stone location from CT images.

Especially in patients with lower pole kidney stones, renal collecting system anatomy should be considered for predicting SWL outcome. Although several studies have reported the effect of infundibular length and width and infundibulopelvic angle on kidney stone clearance, there was no definitive evidence until recently because those studies had limitations including retrospective design and small patient numbers [17–19]. However, the recent, well controlled, prospective study has shown that an infundibular length ≥ 25 mm is the negative predictor for SWL outcome [20]. CT images can provide the information about renal collecting system anatomy without using contrast medium.

## 4. Skin-to-Stone Distance

Representative studies on the relationship between skin-to-stone distance (SSD) and SWL outcomes are summarized in Table 1.

SSD was first reported to be a useful predictor of SWL outcome by Pareek et al. (2005) [21]. In their retrospective study, which targeted 64 patients with lower pole kidney stones, SSD was calculated by measuring three distances from the center of the stone to the skin (0°, 45°, and 90° angles) on noncontrast CT. They showed that SWL for patients with an SSD > 10 cm is likely to fail. Since then, it has been reported that greater SSD is a significant predictor of SWL failure not only in patients with lower pole kidney stones, but also in patients with kidney stones or ureteral stones [22–25]. On the contrary, several studies have reported no association between SSD and SWL outcome [13, 15, 26, 27]. In their retrospective study of patients with renal stones, Weld et al. (2007) reported that stone location impacted SWL success more than SSD, and SSD could not be applied to all renal stones [15]. Jacobs et al. (2008) reported that the impact of SSD on SWL outcome varied by the type of lithotripter used [27]. Recent studies from Asian countries have also shown no association between SSD and SWL success [28–31]. This might be because the number of morbidly obese patients is relatively small in Asian countries.

Future prospective large-scale studies are required to further evaluate the significance of SSD on SWL outcome and examine whether this variable has different predictive powers based on the stone location, the type of lithotripter, and the degree of obesity.

## 5. Mean Stone Density

Mean stone density (MSD) is the mean CT attenuation value of stones and can represent the stone hardness. El-Nahas et al. (2007) reported that MSD > 1000 Hounsfield units (HU) was a significant independent predictor of SWL failure in their prospective study of patients with renal stones [26]. Perks et al. (2008) showed, in their retrospective study of patients with renal stones, that MSD < 900 HU could predict SWL success [22]. On the basis of these results, patients with MSD > 900-1,000 HU have reportedly less successful SWL results in American Urological Association Guidelines [32, 33]. MSD has also been reported to be important in determining the efficacy of SWL treatment by other studies and is widely recognized as a significant predictor of SWL outcome in clinical practice [13, 25, 34–36].

**Figure 1:**
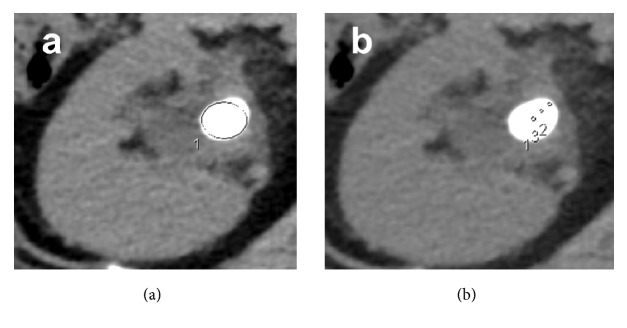
Two techniques used to measure MSD (abdominal window). (a) Elliptical ROI. (b) Average of three ROIs.

However, the cutoff value of MSD is different between the studies, ranging between 593 HU and 1200 HU. One reason may be that the methods for measuring MSD differ between studies. The various methods of measurement of MSD in previous studies are summarized in Table 2. CT image vision depends on the CT window setting, i.e., abdominal windows or bone windows. The measurement of MSD could also vary depending on the method of placement of the region of interest (ROI). In previous studies, MSD has been measured by two main techniques. In one, the elliptical ROI incorporates the stone as a treatment object without including adjacent soft tissue (Figure 1(a)). In the other method, MSD is calculated from three consistent, small, nonoverlapping ROIs chosen for each stone (Figure 1(b)).

As shown in Table 2, MSD measuring methods are different between studies. The recent study has reported that MSD values measured by the various measuring methods were different and the establishment of an accurate and reproducible method for measuring MSD is necessary [37]. To utilize MSD more efficiently, large-scale prospective studies are required. After an appropriate method of measuring of MSD has been ascertained, the optimal cutoff value must be decided.

## 6. Stone Heterogeneity Index/Variation Coefficient of Stone Density

Zarse et al. (2007) reported that the internal structure of calcium oxalate monohydrate stones on CT images could predict lithotripsy fragility in vitro [38]. In addition, Kim et al. (2007) reported a correlation between stone structure and morphology of cystine stones on CT images, and fragility by SWL [39]. The results indicate that stone heterogeneity can affect SWL outcome.

Recently, stone heterogeneity index (SHI), i.e., deviation of stone density, has been reported to be an independent predictor of SWL outcome in patients with ureteral calculi in a large retrospective study (Lee et al., 2016) [30]. Standard deviation is generally used to quantify the amount of variation or dispersion of data values. They reviewed 604 patients with radiopaque ureteral calculi and investigated whether SHI affects the treatment outcome. Two weeks after a single SWL session, treatment success was defined as either stone-free or clinically insignificant, with asymptomatic, residual fragments ≤ 3 mm in the largest stone diameter. Multivariate logistic regression analyses revealed that higher SHI was an independent predictor of treatment success. SHI was concluded to be a useful clinical parameter for stone fragility.

We reported (2017) variation coefficient of stone density (VCSD) as a new predictive parameter associated with stone heterogeneity [31]. Variation coefficient is the standard deviation divided by the mean value. It is generally used to compare dispersion between multiple groups with different average values. We reviewed 245 patients with upper urinary tract calculi who had undergone SWL and compared the predictive powers of MSD, SHI, and VCSD for SWL success. We defined treatment success as stone-free or clinically insignificant residual fragments < 4 mm at maximum diameter within three months following a single SWL session. On receiver operating characteristic curves for treatment success, area under curve of VCSD was larger than that of MSD and SHI. Multivariate logistic regression analysis additionally revealed that VCSD was an independent significant predictor of SWL success in both kidney and ureteral calculi.

Future large-scale prospective studies are required to ascertain the usefulness of SHI and VCSD for predicting SWL outcome.

## 7. CT Texture Analysis

Texture analysis (TA) is a new method of image analysis. This method refers to the characterization of regions in an image by their texture content and attempts to quantify intuitive qualities described by terms such as entropy, kurtosis, and skewness as a function of the spatial variation in pixel intensities. In their ex vivo study, Cui et al. (2017) showed that CT TA metrics entropy and kurtosis could strongly predict fragmentation by SWL [40]. Moreover, TA features identified by machine learning provide incremental accuracy to predict SWL outcomes, according to Mannil et al. (2018) in their preliminary retrospective study targeting 224 patients with untreated kidney stones. [41]. If TA software becomes widely used in the future, it might be useful in clinical practice for prediction of SWL outcome.

## 8. Conclusion

With the advancement in CT technology, various factors for predicting SWL outcome have been reported, including SSD, MSD, SHI, and VCSD. In addition, a new method of image analysis, CT TA, has been developed. Information from CT images could be used effectively to make a suitable treatment plan for patients with upper urinary tract calculi.

## Figures and Tables

**Table 1:** Review of the literature on the relationship between skin-to-stone distance and shock wave lithotripsy outcomes.

Reference	Year	Country	Number of patients	Stone location	Predictive power
Pareek et al. [4]	2005	USA	64	Lower pole	Yes
El-Nahas et al. [9]	2007	Egypt	120	Kidney	No
Weld et al. [10]	2007	USA	200	Kidney	No
Perks et al. [5]	2008	Canada	111	Kidney	Yes
Jacobs et al. [11]	2008	USA	85	Kidney and ureter	No
Bandi et al. [12]	2008	USA	94	Kidney and ureter	No
Ng et al. [6]	2009	Hong Kong	94	Proximal ureter	Yes
Patel et al. [7]	2009	USA	83	Kidney	Yes
Wiesenthal et al. [8]	2010	Canada	422	Kidney and ureter	Yes
Choi et al. [13]	2012	Korea	153	Ureter	No
Tanaka et al. [14]	2013	Japan	75	Kidney and ureter	No
Lee et al. [15]	2016	Korea	604	Ureter	No
Yamashita et al. [16]	2017	Japan	239	Kidney and ureter	No

**Table 2:** Measuring method of mean stone density in previous studies.

Reference	Year	Number of patients	Measuring method			
			CT windows		ROI placement	
			Abdominal	Bone	Elliptical ROI	Three ROIs
El-Nahas et al. [9]	2007	120		○	○	
Perks et al. [19]	2007	76	N/A			○
Perks et al. [5]	2008	111		○	○	○
Kacker et al. [20]	2008	325		○	○	
Bandi et al. [12]	2009	94	○		N/A	
El-Gamal et al. [21]	2009	76		○	N/A	
Wiesenthal et al. [8]	2010	422		○	○	

## Abbreviations

CT: Computed tomography
KUB: Plain abdominal film
US: Ultrasonography
HU: Hounsfield units
MSD: Mean stone density
ROI: Region of interest
SHI: Stone heterogeneity index
SSD: Skin-to-stone distance
SWL: Shock wave lithotripsy
TA: Texture analysis
VCSD: Variation coefficient of stone density.
